# An African grassland responds similarly to long-term fertilization to the Park Grass experiment

**DOI:** 10.1371/journal.pone.0177208

**Published:** 2017-05-11

**Authors:** David Ward, Kevin Kirkman, Zivanai Tsvuura

**Affiliations:** School of Life Sciences, University of KwaZulu-Natal, Scottsville, South Africa; Estacion Experimental de Zonas Aridas, SPAIN

## Abstract

We compared the results of a long-term (65 years) experiment in a South African grassland with the world’s longest-running ecological experiment, the Park Grass study at Rothamsted, U.K. The climate is warm and humid in South Africa and cool and temperate in England. The African grassland has been fertilized with two forms of nitrogen applied at four levels, phosphorus and lime in a crossed design in 96 plots. In 1951, about 84% of plant cover consisted of *Themeda triandra*, *Tristachya leucothrix* and *Setaria nigrirostris*. Currently, the dominant species are *Panicum maximum*, *Setaria sphacelata* and *Eragrostis curvula*, making up 71% of total biomass. As in the Park Grass experiment, we found a significant (additive) interaction effect on ANPP of nitrogen and phosphorus, and a (marginally significant) negative correlation between ANPP and species richness. Unlike the Park Grass experiment, there was no correlation between ANPP and species richness when pH was included as a covariate. There was also a significant negative effect of nitrogen amount and nitrogen form and a positive effect of lime on species richness and species diversity. Soil pH had an important effect on species richness. Liming was insufficient to balance the negative effects on species richness of nitrogen fertilization. There was a significant effect of pH on biomass of three abundant species. There were also significant effects of light on the biomass of four species, with only *Panicum maximum* having a negative response to light. In all of the abundant species, adding total species richness and ANPP to the model increased the amount of variance explained. The biomass of *Eragrostis curvula* and *P*. *maximum* were negatively correlated with species richness while three other abundant species increased with species richness, suggesting that competition and facilitation were active. Consistent with the results from the Park Grass and other long-term fertilization experiments of grasslands, we found a positive effect of soil pH and a negative effect of nitrogen amount on species richness, a more acutely negative effect on species richness of acidic ammonium sulphate fertilizer than limestone ammonium nitrate, a negative relationship between species richness and biomass, and a positive effect on species richness of lime interacting with nitrogen.

## Introduction

Priority effects may play a large role in plant communities [1–4]. The relationships among functional traits, composition and diversity in short-term studies may not reflect vegetation processes in the long-term, because traits of the initial dominants may be unrelated to the long-term outcome of competition [2]. Such priority effects can arise from a variety of factors, including changes in sward structure, litter levels and light availability [5].

One of the ways to minimize priority effects is to conduct long-term experiments. Long-term experiments of rigorous statistical design that have manipulated many of the major nutrients known to be most limiting to plants are rare [6–11]. The longest-running experiment still being conducted is the Park Grass fertilization experiment at Rothamsted in the United Kingdom, which was started in 1856 [12]. Although there are a number of problems with analysis of the Park Grass experiment, including a lack of adequate replication, a large number of fascinating results have been reported from this study, including empirical (e.g., [9, 13–18]) and theoretical outputs (e.g., [6, 9, 19]), and a better understanding of the genetics of local adaptation (e.g., [20–24]). While much of this research has focused on the dynamics and stability of grass communities at Park Grass in response to different forms of nitrogen fertilization (e.g., [9, 14, 15, 18, 25, 26]), some of these papers have focused on the responses of the Park Grass experiment to different fertilizer combinations (e.g., [9, 15, 18]). Some of the most important results from these last-mentioned studies include the additive response of biomass to fertilization with nitrogen and phosphorus, a negative relationship between species richness and biomass, a negative relationship between species richness and level of nitrogen fertilization, a more negative influence of acidic ammonium sulphate than sodium nitrate fertilization, a strong influence of soil pH on species richness, and the positive effect of liming on species richness. Furthermore, Storkey et al. [27] found that the number of species occurring on plots that stopped receiving N fertiliser in 1989 recovered from the negative effects of N fertilization, which was facilitated by liming. While some of these results are not unique to the Park Grass experiment [7, 10, 11, 28–38], seldom have all the variables been tested in a single study, and most of these experiments [10, 33, 37, 39] were based on a far shorter time-frame of experimentation.

In South Africa, a Veld (≈Field) Fertilization Experiment (henceforth, VFE) was established in 1951 on Ukulinga, the University of KwaZulu-Natal’s research farm in Pietermaritzburg (KwaZulu-Natal province on the eastern seaboard of South Africa). This experiment, which is still running, set out to establish the effects of fertilization with nitrogen (two forms: limestone ammonium nitrate (LAN) and ammonium sulphate), four levels of nitrogen fertilization (control; 7.1; 14.1; 21.2 g m^-2^), phosphorus (control; 33.6 g m^-2^) and lime application (control; 225 g m^-2^). Unfortunately, relatively few papers have been published from the 65 years that the experiment has been active [2, 8, 40–47]. Because many of the same factors were examined at Ukulinga as in the Park Grass experiment at Rothamsted, we set out to establish whether similar results occurred in South Africa, despite the fact that there are large differences in climate between the cool, temperate English and warm, humid South African ecosystems. At Rothamsted, the mean annual temperature is ca. 10°C and mean annual rainfall ca. 720 mm, with rainfall almost evenly distributed throughout the year. At Ukulinga, the mean annual temperature is ca. 18°C (mean monthly high temperature is ca. 26°C and mean monthly low temperature is ca. 11°C) and mean annual rainfall is ca. 790 mm with most rainfall concentrated in thunderstorms during the hot summer (Nov-Mar) months (Table 1).

**Table 1:** Comparison of the current study (VFE) with the Park Grass experiment. Details of the Park Grass experiment as from [9].

Name of Experiment	Year Started	Plot Size	No. Plots	Fertilizer	Climate	Precipitation	Mean Annual Temperature
Veld Fertilization Experiment (VFE)	1951	9.0 x 2.7 m	96	nitrate-N, ammonium-N, P, Lime	Warm, humid	790 mm, mostly in summer (November-March)	18°C
Park Grass Experiment	1856	ca. 200 m^2^ (sub-plots range from 75–634 m^2^)	98	Inorganic fertilizers: P, K, Mg, Na, nitrate-N, ammonium-N and Si. Organic manures: farmyard manure and fishmeal (replaced with poultry manure in 2003), straw, sawdust, Lime.	Cool, temperate	720 mm, throughout the year	10°C

We hypothesize that there will be a negative effect of fertilization on species richness and a positive effect on biomass (as measured by Above-ground Net Primary Productivity (ANPP)). We further hypothesize that there will be a more negative effect of acidic N fertilizer and positive effect of liming (and pH) on species richness. We set out to test whether the following results obtained from the Park Grass experiment also occurred at Ukulinga, South Africa:

1. An additive response of biomass to fertilization with nitrogen and phosphorus.
2. A negative relationship between species richness and level of nitrogen fertilization.
3. A more negative effect on species richness of nitrogen fertilization with the more acidic ammonium sulphate than limestone ammonium nitrate.
4. A negative relationship between species richness and biomass (as measured by Above-ground Net Primary Productivity (ANPP)).
5. A positive effect of soil pH on species richness.
6. A positive effect of liming on species richness.

We also attempted to establish how the relationships of the seven most abundant species (*Themeda triandra*, *Tristachya leucothrix*, *Setaria sphacelata*, *Eragrostis curvula*, *E*. *plana*, *Panicum maximum*, *Aristida junciformis*) were affected by these fertilizers. Furthermore, we also measured a number of soil variables (total soil nitrogen, soil respiration, pH, organic carbon) as well as photosynthetically active radiation (PAR) just above the substrate, and attempted to determine their relative importance on these seven species.

## Materials and methods

### Study system and experimental set-up

The VFE is situated at Ukulinga, a research farm of the University of KwaZulu-Natal, Pietermaritzburg, South Africa (29° 24’E, 30° 24’S). The experiment is situated on top of a small plateau at about 840 m a.s.l. Soil is fine-textured and derived from shales. Soil is classified as Westleigh form [48]. The vegetation of the area is classified as southern tall grassveld [49] or, at a larger spatial scale, KwaZulu-Natal hinterland thornveld [50], which is an open savanna of *Acacia (now Vachellia) sieberiana* DC with patches of *Hyparrhenia hirta* L. and other herbaceous species. Native grasses such as *Aristida junciformis* are common in the absence of fire. With regular burning, as is the case on the escarpment at Ukulinga, trees are sparse and *Themeda triandra* is the dominant grass, with *Tristachya leucothrix* and *Heteropogon contortus* also being common [8]. In the VFE plots, the species currently occurring most frequently were *Setaria sphacelata* (89% of plots), *Eragrostis curvula* (74%), *Tristachya leucothrix* (69%), *Themeda triandra* (67%), *Eragrostis plana* (55%) and *Panicum maximum* (49%). Together these species account for much of the herbaceous ANPP [45, 46]. The native grass species in the locality all use the C_4_ photosynthetic pathway [45]. There has been no grazing on the experimental site for >65 yrs.

This experiment started in 1951, manipulating nitrogen, phosphorus and lime. There are 96 plots, each in 9.0 x 2.7 m size with 1 m spacing between plots [42]. A full description of the VFE, which was established on virgin native grassland, is given elsewhere [40, 41, 42, 46]. This experiment was replicated in three blocks of 32 plots each, giving a 4 x 2^3^ factorial design. Two forms of nitrogen were applied, limestone ammonium nitrate (LAN) and ammonium sulphate (NH_4_). Four levels of nitrogen fertilizer were applied annually in this experiment, viz. (0 (i.e. control), 7.1, 14.1 and 21.2 g m^-2^), with the same amount of N being applied in both the limestone ammonium nitrate (LAN) and ammonium sulphate treatments [2]. Each form of N was not applied in combination with the other, but only with P and lime. Phosphorus was applied annually as super-phosphate at two levels (0 (control) and 33.6 g m^-2^). Lime treatments were applied every five years at two levels (0 (control) and 225 g m^-2^).

### Soil measurements

We took five soil samples per plot and then bulked them in a single sample for analysis of soil quality for each plot (see details below). We determined total nitrogen using an Elementar^®^ Rapid N Cube nitrogen analyzer. This analyzer uses the Dumas combustion method of nitrogen analysis. To measure soil carbon dioxide respiration (measured in mg CO_2_-C kg^-1^ soil), hereafter abbreviated as *soil respiration*, we used the Solvita^®^ gel system. A pH-sensitive gel (paddle) narrows to a point that can be pushed into the soil [51, 52]. The procedure is standardized with a special soil-capillary moistening beaker to allow the correct amount of water infiltration for the test. After 24 h, the paddle is removed from the incubation jar and analyzed with a digital color reader (DCR). Additional variables measured in the VFE were pH and organic carbon (measured as mass loss upon ignition). For further details, see [47].

### Phytomass and species composition

We measured the aboveground phytomass production (ANPP) of entire plots using a modification of the dry-weight-rank method [53]. These measurements were carried out in May 2010 and between January and February 2011. The May 2010 measures occurred on the regrowth that arose from the mowing of the grass in December 2009, while the February 2011 measurements were on the regrowth arising from the grass mowed in August 2010 [47]. We used aboveground net primary production (ANPP) for ease of comparison with previous studies on the same experiment, and because ANPP has been shown to be a good predictor of competitive ability [54]. We also measured species richness for each plot and recorded ANPP by weighing the dry material. For further details, see [46]. Simple plant traits such as plant height and leaf width have been shown to be important indices of competition among species [2, 54–58]. We use these here in an attempt to establish the potential role of competition on the aboveground phytomass of the seven most abundant species (*Themeda triandra*, *Tristachya leucothrix*, *Setaria sphacelata*, *Eragrostis curvula*, *E*. *plana*, *Panicum maximum*, *Aristida junciformis*) in response to fertilization with nitrogen, phosphorus and lime. Data are available at doi:10.5061/dryad.f13r4.

### Statistical analyses

The effects of the nutrient treatments on ANPP, species richness and species diversity were compared using generalized linear models with the nutrient and lime applications and block as the main factors. We performed these analyses on total ANPP, species richness and species diversity of all species and then individually for the common species (listed above). We tested for collinearity and multicollinearity in each case [59]. We also included soil nitrogen, soil respiration, pH, light (measured as photosynthetically active radiation (PAR, %) with a Decagon Sunfleck ceptometer) and organic carbon as covariates, and also included species richness and ANPP in analyses of the common species. For each of the main species, the data were square-root transformed to normalize residuals before analyses while no transformations were necessary for total ANPP, species richness and species diversity data. Where a factor with multiple levels was found to be significant (e.g. nitrogen form), we used LSD *post hoc* tests to differentiate among treatments. All analyses were undertaken using SPSS v. 24 [60].

When differentiating among models, we use Akaike’s information criterion (AIC) [61]. We computed AIC for each of the several models and selected the model with the smallest AIC value as “best” [62–64]. Following the approach of Burnham et al. [65], we calculated the ΔAIC values for each hypothesis and selected the one with the smallest information loss or “smallest distance from full reality” as the best hypothesis and obtained a ranking of the rest of the models. Although a minimum acceptable ΔAIC is often construed as 2, 2–6 should not be excluded [63, 64].

## Results

### Above-ground biomass (ANPP)

**Fig 1:**
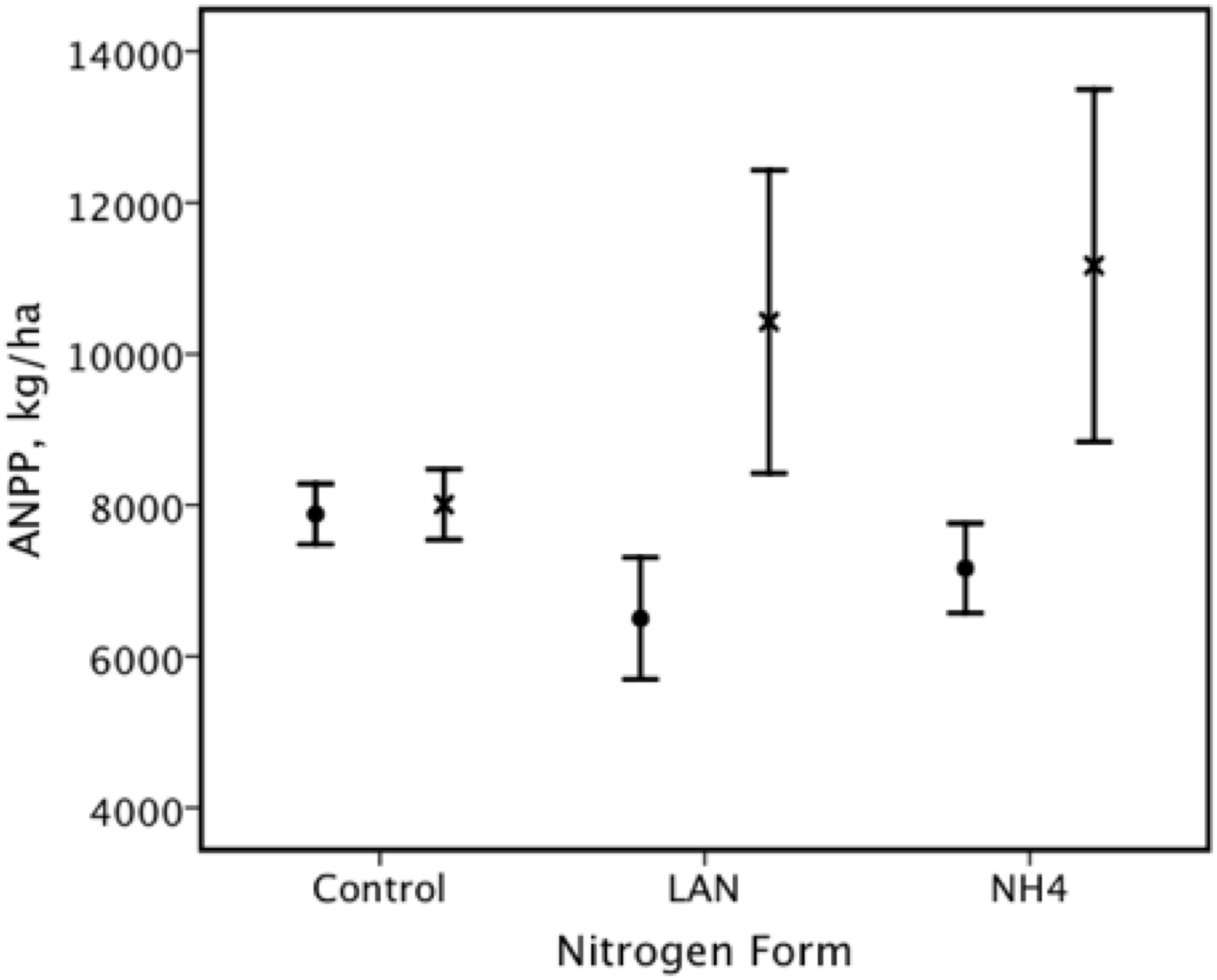
There was a significant interaction effect between nitrogen form and phosphorus fertilization on mean (± 95% C.I.) Aboveground Net Primary Production (ANPP). Nitrogen forms were control, limestone ammonium nitrate (LAN) and ammonium sulphate (NH4). Control phosphorus is indicated by solid circles (•) and the phosphorus fertilization by X.

**Fig 2:**
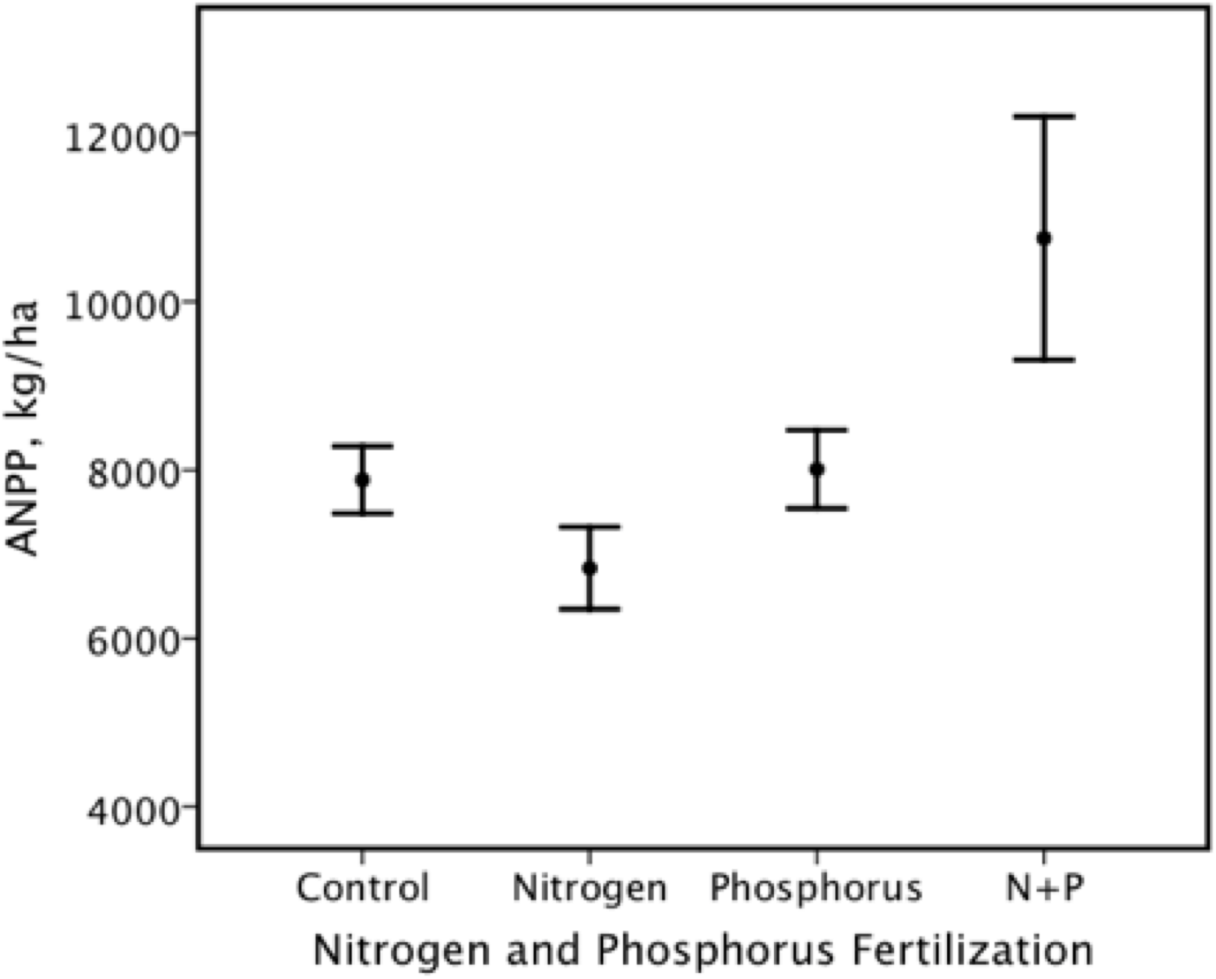
There was a significant additive effect on mean (± 95% C.I.) Aboveground Net Primary Production (ANPP) of nitrogen and phosphorus fertilization. N+P = nitrogen + phosphorus fertilization.

In the test of the effects of nitrogen form, nitrogen level, phosphorus and lime on Aboveground Net Primary Production (ANPP), the overall effect was significant; this was due to the significant effect of phosphorus only (Table 2a). There was also a significant nitrogen X phosphorus interaction (Table 2a, Fig 1). We found that there was a significant additive effect of nitrogen and phosphorus fertilization (Fig 2).

**Table 2:** Generalized linear model analyses of effects of fertilizer. These fertilizers are nitrogen form (control, limestone ammonium nitrate (LAN) and ammonium sulphate), nitrogen level (0 (control), 7.1, 14.1 and 21.2 g m^-2^), phosphorus (0 (control), 33.6 g m^-2^) and lime (0 (control), 225 g m^-2^) on (a) Aboveground Net Primary Production (ANPP), (b) species richness, and (c) species diversity (Shannon-Wiener H’). Nitrogen level was nested {} within nitrogen form.

**a) Dependent variable: ANPP**		
**Factor**	**Wald’s χ**^**2**^	**p**
Nitrogen form	3.217	0.200
Nitrogen level{Nitrogen form}	6.025	0.197
Phosphorus	**24.686**	<0.001*
Lime	0.032	0.858
Nitrogen*Phosphorus	**9.864**	0.007*
Nitrogen*Lime	1.055	0.590
Phosphorus*Lime	0.225	0.635
Nitrogen*Phosphorus*Lime	2.693	0.260
Block	0.617	0.735
**b) Dependent variable: Species Richness**		
**Factor**	**Wald’s χ**^**2**^	**p**
Nitrogen form	**190.797**	<0.001*
Nitrogen level{Nitrogen form}	**74.747**	<0.001*
Phosphorus	0.209	0.648
Lime	**37.560**	<0.001*
Nitrogen*Phosphorus	0.183	0.913
Nitrogen*Lime	**14.879**	0.001*
Phosphorus*Lime	0.986	0.321
Nitrogen*Phosphorus*Lime	**8.315**	0.016*
Block	**6.388**	0.041*
**c) Dependent variable: Species Diversity**		
**Factor**	**Wald’s χ**^**2**^	**p**
Nitrogen form	**23.298**	<0.001*
Nitrogen level{Nitrogen form}	**16.989**	0.002*
Phosphorus	0.638	0.424
Lime	**6.956**	0.008*
Nitrogen*Phosphorus	2.372	0.305
Nitrogen*Lime	**7.087**	0.029*
Phosphorus*Lime	0.750	0.386
Nitrogen*Phosphorus*Lime	3.275	0.194
Block	5.884	0.053

* = significant difference.

The most significant improvement in the model occurred with species richness and species diversity added as covariates (ΔAIC = 27.0). With all soil parameters included (i.e. pH, soil respiration, nitrogen, organic carbon, as well as species richness and species diversity), ΔAIC was 26.5. Removing all parameters except species diversity, ΔAIC was 26.5.

We found no significant relationship between ANPP and total soil nitrogen (r = 0.03; F = 0.076; p = 0.783) or between ANPP and organic carbon (r = 0.01; F = 0.007; p = 0.934). Similarly, there was no significant effect of pH on ANPP (r = 0.05; F = 0.252, p = 0.617).

When ANPP was separately regressed on species richness, there was a marginally significant (negative) relationship (r = -0.20; F = 3.756, p = 0.056). We also tested for a relationship between ANPP and species richness when we controlled for pH (covariate). We found no significant relationship (F = 1.06; p = 0.444). There was also no significant relationship between ANPP and species diversity (r = 0.16; F = 2.59; p = 0.111).

### Species richness

**Fig 3:**
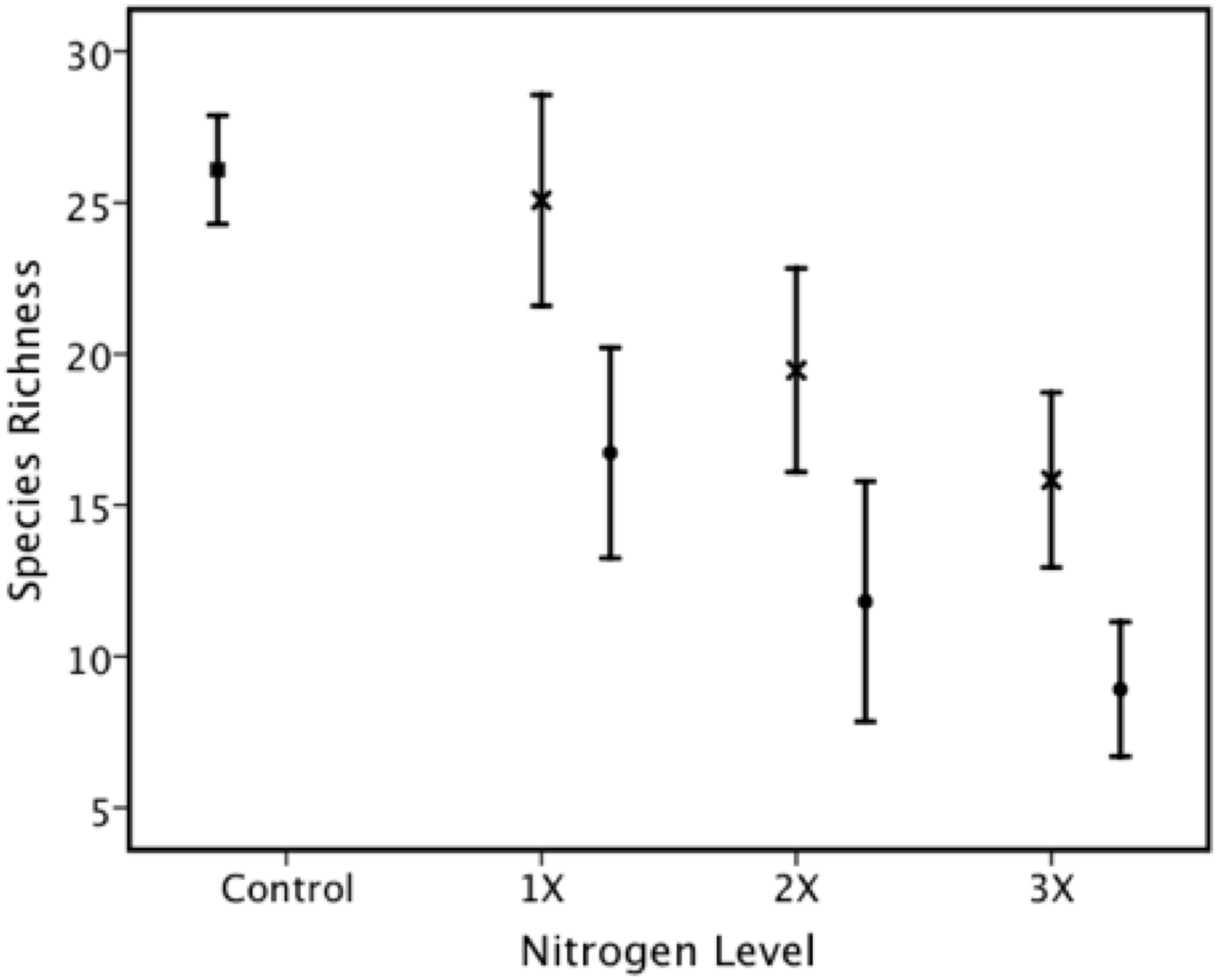
There was a significant effect of nitrogen form and level of nitrogen application on species richness. There was a more negative effect on species richness of acidic ammonium sulphate fertilization (NH4—solid circles •) with increasing level of fertilization than for LAN (X). Highest species richness was for the control (no treatment—open square □).

**Fig 4:**
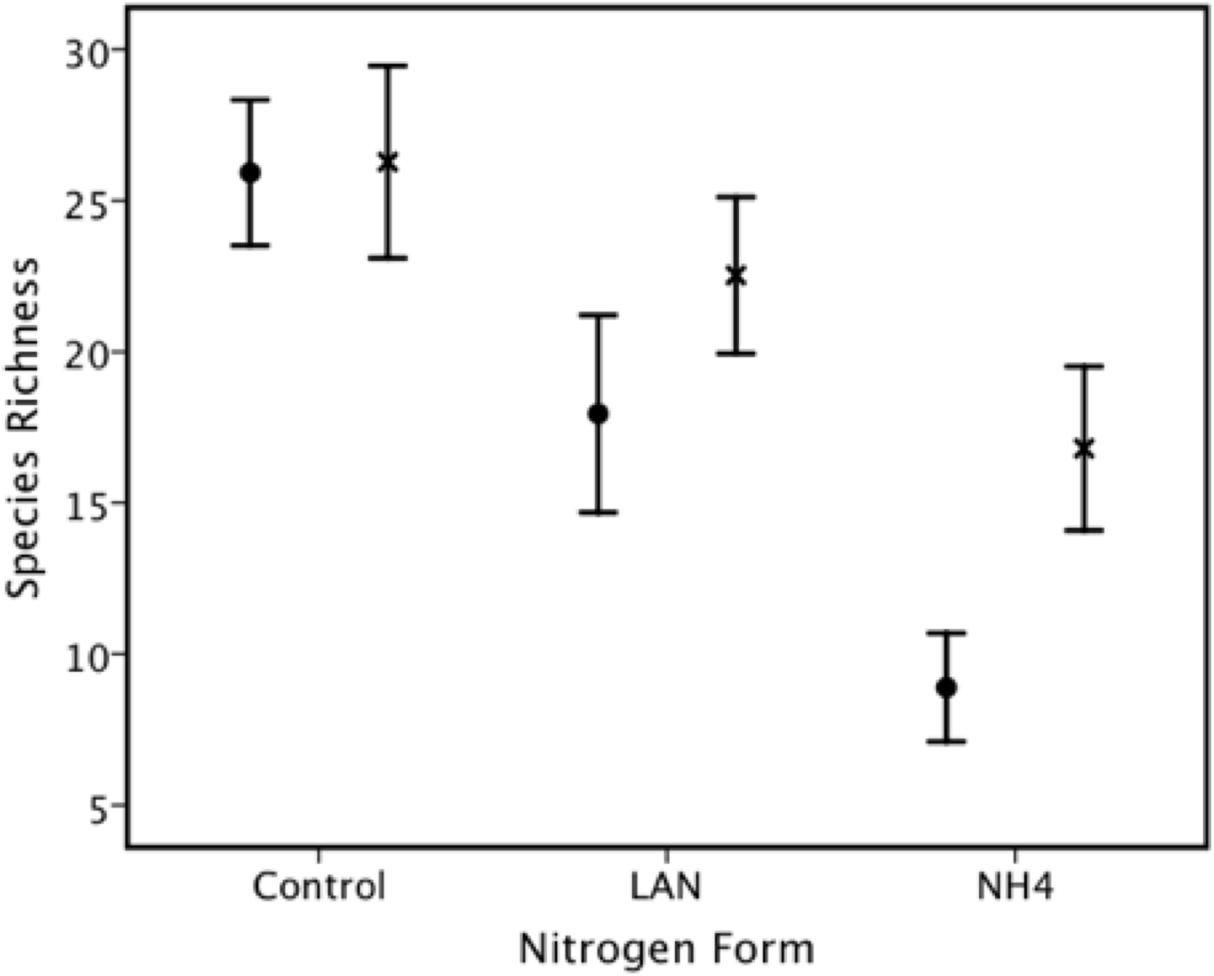
There was a significant interaction effect between nitrogen form and lime application on species richness. The effect of lime (X) was to increase species richness relative to the nitrogen form (particularly noticeable with the ammonium sulphate (NH4) fertilization). However, the control (no treatment) had the highest species richness. LAN = limestone ammonium nitrate. Control lime is indicated by solid circles (•).

Species richness ranged from 5 to 36, with mean ± S.E. = 18.9 ± 0.80. We found a significant effect on species richness of nitrogen form (ammonium sulphate and LAN), nitrogen level (Fig 3) and lime (Table 2b) and a significant nitrogen form X lime interaction (Fig 4).

In the test of the effects of nitrogen form, nitrogen level, phosphorus and lime on species richness, the overall effect was significant, and this was due to the significant effect of nitrogen form, nitrogen level and lime, but there was no significant effect of phosphorus fertilization (Table 2b). The best-fit model in terms of ΔAIC over the fertilizers only was using species diversity and ANPP as covariates (ΔAIC = 9.0), although this was little improved over the inclusion of species diversity only in addition to the fertilizers (ΔAIC = 7.8) or including all soil variables (soil nitrogen, soil respiration, pH, organic carbon) in addition to species diversity and ANPP (ΔAIC = 7.9). In an LSD *post hoc* test, there was no significant difference in the effects on species richness between lime application and the control, but nitrogen fertilization resulted in fewer species, with a significant increase in species richness for lime (but still considerably less than the control or lime only) (Fig 4).

**Fig 5:**
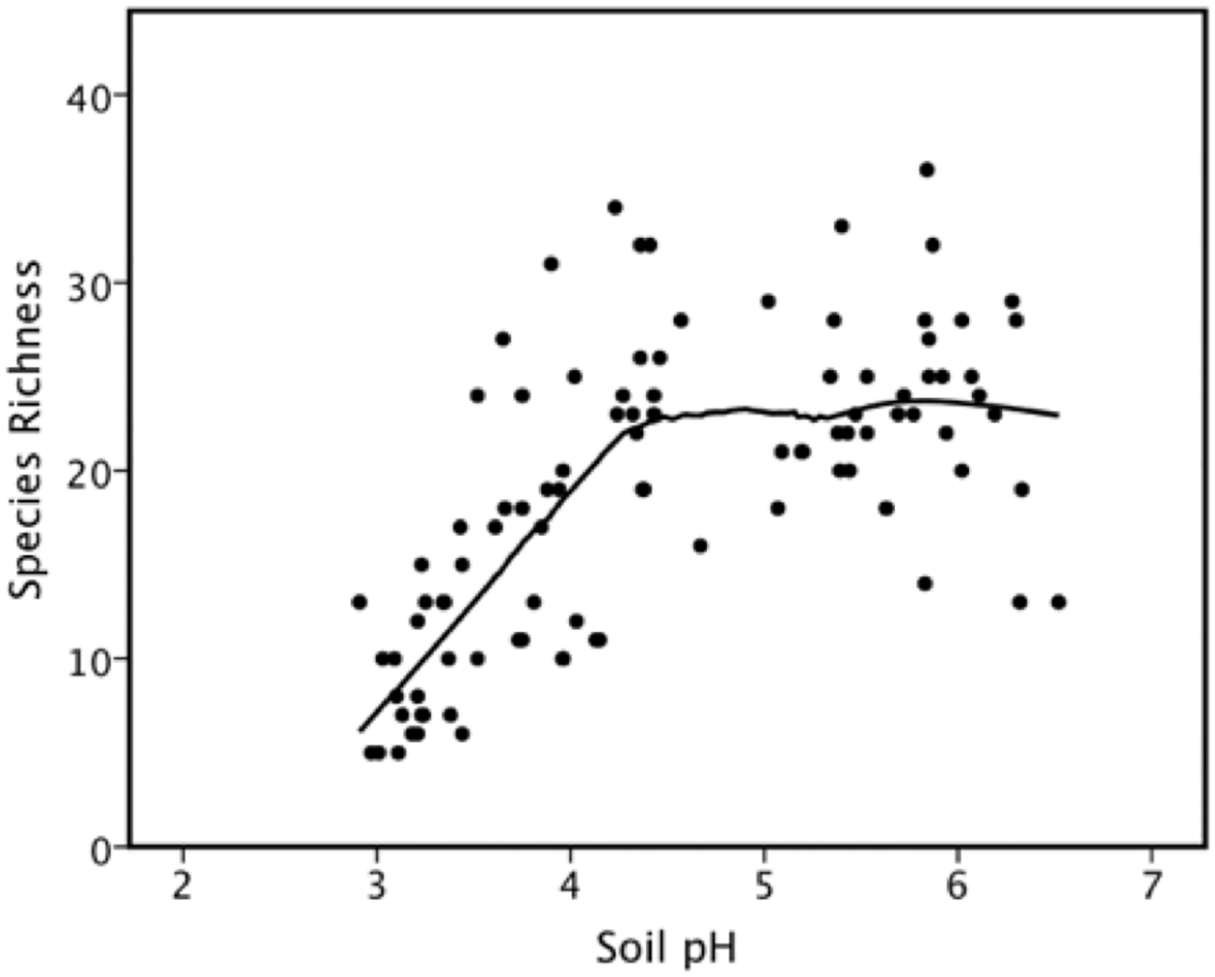
There was a significant positive relationship between species richness and soil pH. However, this relationship was better explained by a piecewise regression. We have used a LOWESS plot here, with a threshold at a pH of about 4.5.

There was a significant positive correlation between species richness and pH (r = 0.62; F = 58.04; p < 0.001). A quadratic regression explained the variance better than the simple linear regression (r^2^ = 0.74 (quadratic) vs. 0.62 (linear); ΔAIC = 26.6). The threshold is at about 5.3. This significant relationship appeared to be better explained by a piecewise regression (Fig 5). The locally weighted scatterplot smoothing (LOWESS or loess) regression [66, 67] showed that the threshold was at about 4.5.

There was a significant negative correlation between species richness and total soil nitrogen (r = -0.45; F = 23.48; P < 0.001), and a significant positive correlation between species richness and soil respiration (r = 0.57; F = 45.47, p < 0.001). We have shown elsewhere that there was a negative correlation between total soil nitrogen and soil respiration [47], so it is unclear whether species richness declined as a consequence of nitrogen fertilization or increased due to higher soil respiration. However, in a multiple regression with both soil nitrogen and soil respiration as independent variables, the (absolute) value of the standardized (β) coefficient was higher for soil respiration (0.498) than for soil nitrogen (-0.340) (both p values < 0.001), indicating that soil respiration was the most important variable.

### Species diversity

In the test of the effects of nitrogen form, nitrogen level, phosphorus and lime on species diversity, the overall effect on species diversity was significant, and this was due to the significant effect of nitrogen form, nitrogen level and lime, but there was no significant effect of phosphorus (Table 2c). Thus, these results are similar to those for species richness but not ANPP. The best-fit model in terms of ΔAIC over the model for fertilizers only was using species richness and ANPP as covariates (ΔAIC = 17.1).

There was a significant positive correlation between species diversity and total soil nitrogen (r = 0.30; F = 9.08; p = 0.003). There was a significant positive correlation with pH (r = 0.40; F = 17.31; p < 0.001). There was a significant negative correlation between species diversity and species richness (r = -0.57; F = 43.99; p < 0.001).

### Individual species’ responses

We tested the effects of fertilizers on the aboveground phytomass of the seven most abundant species (*Themeda triandra*, *Tristachya leucothrix*, *Setaria sphacelata*, *Eragrostis curvula*, *E*. *plana*, *Panicum maximum*, *Aristida junciformis*) (Table 3).

**Table 3:** Generalized linear model analysis of fertilizers on the phytomass of the seven most abundant species. We measured the effects of nitrogen form (Control, LAN and ammonium sulphate), nitrogen level (0, 7.1, 14.1 and 21.2 g m^-2^), phosphorus (0 (control), 33.6 g m^-2^) and lime (0 (control), 225 g m^-2^) and their interactions on phytomass (k ha^-1^) of the seven most abundant grass species, *Themeda triandra*, *Tristachya leucothrix*, *Setaria sphacelata*, *Eragrostis curvula*, *E*. *plana*, *Panicum maximum* and *Aristida junciformis*. {} = nested factor. χ^2^ = Wald’s χ^2^.

	*Themeda triandra*		*Tristachya leucothrix*		*Setaria sphacelata*		*Eragrostis curvula*		*Eragrostis plana*		*Panicum maximum*		*Aristida junciformis*	
Factor	χ^2^	p	χ^2^	p	χ^2^	p	χ^2^	p	χ^2^	p	χ^2^	p	χ^2^	p
N form	**479.284**	<0.001*	**110.558**	<0.001*	**10.629**	0.005*	**67.708**	<0.001*	**34.192**	<0.001*	**60.187**	<0.001*	5.107	0.078
N level{N form}	**47.304**	<0.001*	**98.256**	<0.001*	**42.869**	<0.001*	5.472	0.242	6.931	0.140	**42.564**	<0.001*	3.060	0.548
P	**16.851**	<0.001*	**30.290**	<0.001*	.194	0.660	**7.399**	0.007*	**5.436**	0.020*	**93.039**	<0.001*	**7.957**	0.005*
Lime	**9.090**	0.003*	**45.653**	<0.001*	**20.453**	<0.001*	**6.450**	0.011*	2.107	0.147	0.002	0.962	3.795	0.051
N*P	0.906	0.636	4.197	0.123	3.032	0.220	**6.272**	0.043*	4.334	0.115	**38.769**	<0.001*	3.728	0.155
N*Lime	4.714	0.095	**27.219**	<0.001*	2.928	0.231	5.715	0.057	1.371	0.504	0.881	0.644	3.604	0.165
P*Lime	0.349	0.555	0.021	0.886	**4.245**	**0.039**	0.541	0.462	0.039	0.843	0.373	0.541	**4.116**	0.042*
N*P*Lime	**19.164**	<0.001*	1.385	0.500	2.904	0.234	2.579	0.275	0.969	0.616	3.096	0.213	5.361	0.069

* = significant difference.

For four of the species (*T*. *triandra*, *T*. *leucothrix*, *S*. *sphacelata*, *P*. *maximum*), there were significant effects of nitrogen form and nitrogen level. For six species (*T*. *triandra*, *T*. *leucothrix*, *E*. *curvula*, *E*. *plana*, *P*. *maximum*, *A*. *junciformis*) there was a significant effect of phosphorus (i.e. only *S*. *sphacelata* had a non-significant effect for P). Lime application was significant for four species (*T*. *triandra*, *T*. *leucothrix*, *S*. *sphacelata* and *E*. *curvula*). For *E*. *curvula* and *P*. *maximum*, there was a significant nitrogen X phosphorus interaction, *T*. *leucothrix* had a significant nitrogen X lime interaction and *S*. *sphacelata* and *A*. *junciformis* had a significant phosphorus X lime interaction.

We further tested the importance of these analyses by comparing ΔAIC for the addition of covariates (soils, photosynthetically active radiation (PAR), species richness and ANPP). For three of the species (*S*. *sphacelata*, *E*. *curvula*, *E*. *plana*), adding fertilizers as well as all soils variables (nitrogen, soil respiration, pH, organic carbon), PAR, species richness and ANPP improved the model based on ΔAIC. For two other species (*T*. *triandra*, *P*. *maximum*), adding species richness and ANPP produced the best-fit model in terms of ΔAIC. For *T*. *leucothrix*, adding species richness and ANPP also produced the best-fit model in terms of ΔAIC (2.5) but it was not much better than fertilizers only. For *A*. *junciformis*, the fertilizers-only model was best.

We ran multiple regressions of the phytomass of the seven most abundant species against soil nitrogen, soil microbial respiration, pH, organic carbon, and photosynthetically active radiation (PAR) (Table 4a). As noted previously [46], the nitrophobic species, *T*. *triandra* and *T*. *leucothrix*, showed significant negative effects of soil nitrogen. For *S*. *sphacelata*, *E*. *curvula* and *P*. *maximum*, there was a significant effect of pH, but the effect for *S*. *sphacelata* was positive (i.e. higher pH led to higher phytomass) while for *E*. *curvula* and *P*. *maximum* the effect was negative. Interestingly, the nitrophilic *P*. *maximum* did not show any significant effect of soil nitrogen but did respond negatively to pH (Table 4a). There were significant effects of PAR for *E*. *curvula*, *E*. *plana*, *P*. *maximum* and *A*. *junciformis*, with only *P*. *maximum* having a negative response to light. We then ran the same multiple regressions with species richness and biomass per plot added to the abovementioned soil and light variables. In all cases, adding species richness and biomass increased the r^2^ value (Table 4a and 4b).

**Table 4:** Multiple regression of the phytomass of the seven most common species against soil and light parameters across all plots. (a) Soil nitrogen, pH, soil microbial respiration, organic carbon and PAR. (b) The same variables as well as species richness and biomass per plot. β values are standardized coefficients. The absolute value of β coefficients are an index of the importance of that variable as long as it is significant. The r^2^ values are coefficients of determination of all the variables. ΔAIC = change in AIC from multiple regression containing soil and light variables only. Note that there is an increase in the r^2^ value in all cases in (b) relative to (a) for each species. ΔAIC < 2 in the cases of *E*. *curvula* and *E*. *plana* (Table 4b), which is conventionally viewed as an insufficient improvement over the models in Table 4a (Burnham & Anderson 2002). PAR = photosynthetically active radiation.

**a)**	*Themeda triandra*		*Tristachya leucothrix*		*Setaria sphacelata*		*Eragrostis curvula*		*Eragrostis plana*		*Panicum maximum*		*Aristida junciformis*	
r^2^	0.23		0.14		0.26		0.29		0.15		0.44		0.12	
**Factor**	**β**	**p**	**β**	**p**	**β**	**p**	**β**	**p**	**β**	**p**	**β**	**p**	**β**	**p**
Total Nitrogen	**-0.268**	0.006*	**-0.230**	0.032*	0.088	0.355	0.061	0.513	0.059	0.574	0.051	0.544	-0.054	0.606
pH	**0.264**	0.025*	-0.174	0.181	**0.545**	<0.001*	**-0.295**	0.011*	-0.105	0.411	**-0.263**	0.012*	-0.168	0.191
Soil respiration	0.168	0.128	0.236	0.055	0.007	0.947	**-0.220**	0.043*	-0.079	0.513	0.047	0.630	-0.166	0.169
Organic Carbon	0.022	0.801	0.170	0.086	-0.020	0.826	0.006	0.943	0.009	0.923	-0.003	0.972	-0.111	0.257
PAR	0.177	0.053	0.162	0.109	-0.091	0.315	**0.259**	0.004*	**0.338**	<0.001*	**-0.664**	<0.001*	**0.220**	0.029*
**b)**	*Themeda triandra*		*Tristachya leucothrix*		*Setaria sphacelata*		*Eragrostis curvula*		*Eragrostis plana*		*Panicum maximum*		*Aristida junciformis*	
r^2^	0.34		0.23		0.35		0.32		0.19		0.81		0.36 (p = 0.086)	
ΔAIC	10.902		5.734		8.170		1.198		0.798		102.132		3.336	
**Factor**	**β**	**p**	**β**	**p**	**β**	**p**	**β**	**p**	**β**	**p**	**β**	**p**	**β**	**p**
Total Nitrogen	-0.122	0.183	-0.138	0.197	**0.201**	0.035*	-0.054	0.566	-0.023	0.830	0.002	0.977	-0.068	0.549
pH	0.073	0.528	**-0.367**	0.007*	**0.352**	0.004*	-0.208	0.082	-0.123	0.364	0.020	0.813	-.0148	0.299
Soil Respiration	-0.012	0.913	0.123	0.325	-0.132	0.231	-0.078	0.475	0.034	0.717	0.110	0.157	-0.150	0.257
Organic Carbon	0.002	0.983	0.165	0.080	-0.031	0.710	0.029	0.723	0.129	0.318	-0.019	0.743	-0.109	0.271
PAR	0.152	0.164	0.016	0.901	-0.192	0.090	0.164	0.147	-0.203	0.144	**-0.267**	0.001*	0.226	0.096
Species Richness	**0.562**	<0.001*	**0.418**	0.003*	**0.474**	<0.001*	**-0.386**	0.002*	**-0.345**	0.008*	**-0.385**	<0.001*	-0.055	0.705
ANPP	0.065	0.550	-0.140	0.269	-0.062	0.579	-0.209	0.063	-0.023	0.830	**0.514**	<0.001*	-0.002	0.990

* = significant difference.

**Fig 6:**
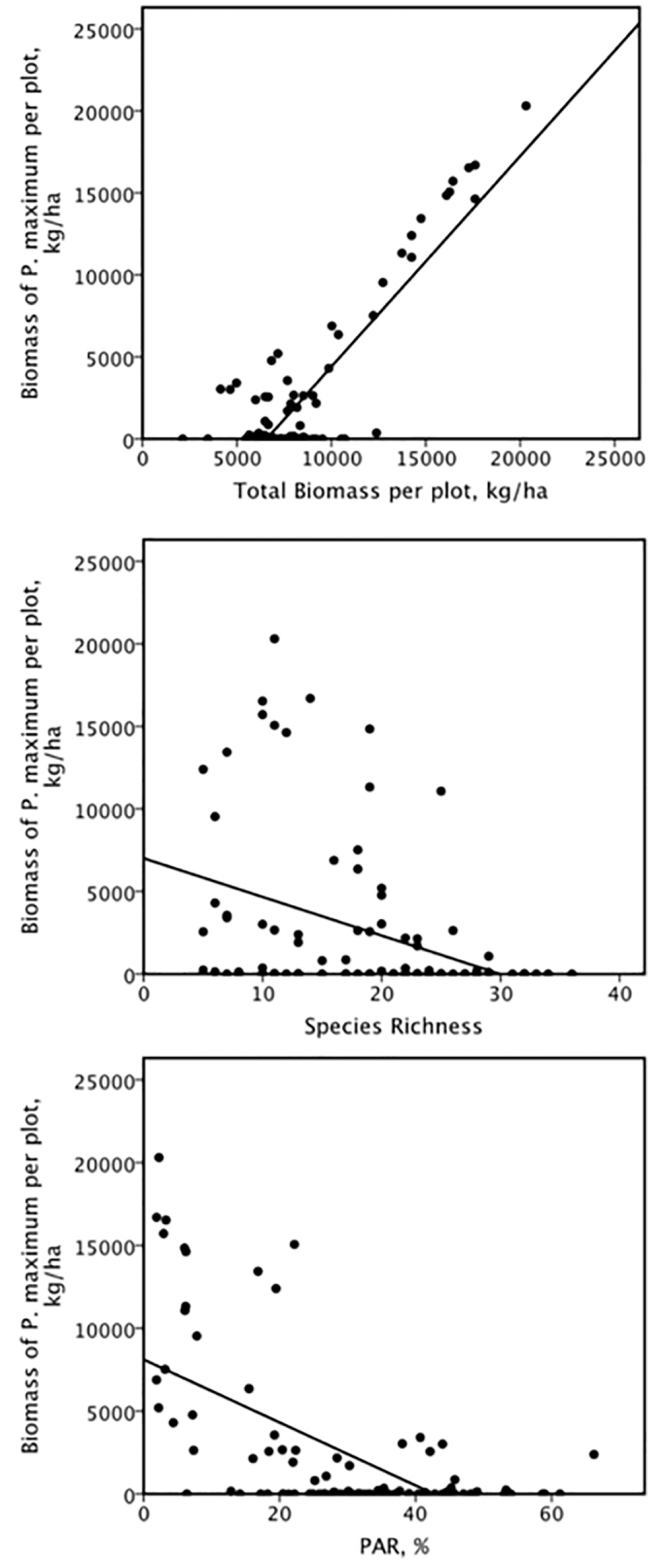
There were significant correlations between phytomass of *Panicum maximum* per plot and ANPP, species richness and photosynthetically active radiation. a) There was a significant positive correlation (r = 0.87) between phytomass of *Panicum maximum* per plot and total biomass (ANPP) across all plots. b) There was a significant negative correlation (r = -0.38) between phytomass of *Panicum maximum* per plot and species richness. c) There was a significant negative correlation (r = -0.61) between phytomass of *Panicum maximum* per plot and photosynthetically active radiation (PAR), measured as % sunlight reaching just above the substrate.

In most cases, species richness was significant but there was no significant effect of any of the soil or light variables (Table 4b), except in the case of *P*. *maximum* where there was a significant (negative) effect of light. For *E*. *curvula*, *E*. *plana* and *P*. *maximum*, there was a significant negative effect of species richness, indicating density dependence, but for *T*. *triandra*, *T*. *leucothrix* and *S*. *sphacelata*, the effect was positive. For *P*. *maximum*, the effects of both biomass and species richness were significant. Most notable was the large increase in the overall variance (r^2^) for *P*. *maximum* due to the significance of ANPP (β = 0.727) and then species richness (β = -0.288). There was a significant positive correlation for *P*. *maximum* biomass with total aboveground biomass per plot (r = 0.87, F = 284.761, p < 0.001) (Fig 6a), a negative correlation with species richness (r = -0.38, F = 15.718, p < 0.001) (Fig 6b) and a negative correlation with PAR (r = -0.61, F = 56.274, p < 0.001) (Fig 6c). The overall multiple regression for *A*. *junciformis* was non-significant (p = 0.084) when species richness and biomass were included (Table 4b) but was significant with the soil and PAR variables only (Table 4a) due to the significant positive effect of PAR (β = 0.209).

## Discussion

### Additive effects of nitrogen and phosphorus on ANPP

There were many similarities between the results obtained in the VFE at Ukulinga and the Park Grass experiment [9, 15, 18], despite large differences in climate. One of the most notable similarities was the additive effects of nitrogen and phosphorus fertilization on ANPP (Table 2; Figs 1 and 2), as previously reported for the Park Grass experiment [15, 18]. Earlier studies of the VFE at Ukulinga [42, 45, 46] had also found that the addition of nitrogen and phosphorus led to the highest yield. Indeed, such co-limitation by nitrogen and phosphorus has been recorded for South African grasslands in general [68]. In a global review of the effects of co-limitation by nitrogen and phosphorus, Elser et al. [35] found that this situation is the norm for both aquatic and terrestrial ecosystems.

Fynn and O’Connor [2] had previously shown that major compositional responses were determined by N- and P-fertilization in the VFE at Ukulinga, which had the greatest effect on ANPP. They also found that the shorter grasses, such as *Themeda triandra* (and *Tristachya leucothrix* in our study) tended to be eliminated with fertilization and replaced by the taller grasses, such as *Panicum maximum* and *Setaria sphacelata*. The mechanism behind this is probably related to access to light. Tsvuura and Kirkman [46] found that fertilization with nitrogen decreased the amount of photosynthetically active radiation (PAR) reaching the ground because it increased the amount of biomass in the plots. This effect was exacerbated by the addition of phosphorus.

### Species richness and level of nitrogen fertilization

The negative effect of nitrogen fertilization at Ukulinga on species richness (Fig 3) was recorded previously at Ukulinga [42]. These authors found that the usually common *Themeda triandra* and *Tristachya leucothrix* were replaced by other grass species due to the application of nitrogen. These two species also declined in response to nitrogen fertilization in the current study (Table 4a). Interestingly, there was no significant response of ANPP to nitrogen form or nitrogen amount (although there was a significant nitrogen X phosphorus interaction) but there was a main effect of phosphorus on ANPP. Contrastingly, there was a significant response of both species richness and species diversity to nitrogen form and amount but no effect of phosphorus on either of the two last-mentioned variables. The decline in species richness with nitrogen fertilization has also been recorded for the Park Grass experiment [9, 14, 15, 18], as it has in other studies in other parts of the world (see e.g., [7, 30, 32–34, 38, 55, 69, 70]).

Crawley et al. [18] also recorded that the effects of fertilization by acidic ammonium sulphate had a more negative effect on species richness than fertilization with sodium nitrate. We had a similar result with our fertilization with ammonium sulphate vs. limestone ammonium nitrate (LAN) (Fig 3). In an earlier study of fertilization at Ukulinga, Le Roux and Mentis [42] did not detect an effect of nitrogen form (LAN vs. ammonium sulphate). However, by the time of subsequent studies [45–47], there was an effect of form of nitrogen as well as level of fertilizer (Fig 3). These results are consistent with the overall result showing a significant negative effect of pH (see also [9, 15, 18])–see further discussion below.

### Species richness and productivity

In the Park Grass experiment, Crawley et al. [18] found that there was a negative relationship between productivity (ANPP) and species richness in their small-scale experimental plots. We found a similar negative relationship between species richness and productivity (ANPP), although the significance (p = 0.056) was marginal. The correlation coefficients of the two studies were very similar (r = -0.22 in the Park Grass experiment; r = -0.20 in our study). However, Tsvuura and Kirkman [46] found that there was a non-significant relationship in one year in the VFE at Ukulinga and a significant relationship in the following year, suggesting that differences in rainfall may have been responsible (see also variance due to rainfall among years in the Park Grass experiment [13, 71]). We note that Fynn and O’Connor [2] found a negative relationship between species composition and ANPP in an earlier study in the VFE at Ukulinga, which they claim was initiated by the effect of *fertilization* on ANPP and not by the effect of *composition* on ANPP because they found that fertilization had a major effect on ANPP after one year of fertilization of the Ukulinga experiment (1951/1952 season) when composition was very similar in all plots.

This experimentally demonstrated negative relationship between species richness and productivity is the opposite of the commonly recorded pattern of a positive or unimodal relationship between species richness and productivity for larger gradients (e.g., [72–75]). Such a negative relationship between species richness and productivity in fertilized plots is usually ascribed to interspecific competition (often for light) occurring under high productivity [15]. Another possible reason for a decline in species richness at high productivity is a passive consequence of a limited pool of species possessing characteristics necessary to survive and outcompete other species in high-fertility environments [76–78]. At a small spatial scale, fertilized plots often show a negative relationship between species richness and productivity because of increasing extinction as productivity increases [79] or because recruitment falls as standing crop biomass increases [80, 81]. There has been a negative relationship between species richness and productivity in the Park Grass experiment recorded in every year since 1862 [15]. This effect is not independent of pH—for any given level of productivity, more acidic plots had fewer species [15]. We found a similar result for the relationship between species richness and soil pH (Fig 5), although using pH as a covariate showed that there was no significant relationship between species richness and productivity (ANPP).

The sample size in our study (n = 96) was similar to that for the Park Grass experiment (n = 97 –[18]). The size of our plots was clearly much smaller (24.3 m^2^) than the Park Grass plots (ca. 200 m^2^). We note that Mittelbach et al. [74] found that sample size and plot size did not affect the probability of finding a particular productivity-diversity relationship (e.g., positive, hump-shaped, negative). The length of time that these experiments have been running should have allowed a relationship (be it positive, negative or unimodal) to develop.

In a review of grassland fertilization experiments run at 48 sites on five continents, Adler et al. [10] considered productivity to be a poor predictor of plant species richness; we note that their plots (25 m^2^) are similar in area to ours. Some [31, 82] contend that even long-term fertilization experiments are not good predictors of the relationship between species richness and productivity because they are relatively small-scale perturbations whereas the pattern of species richness over natural productivity gradients is influenced by long-term ecological (such as dispersal) and evolutionary processes (e.g. speciation).

### Soil pH, liming and species richness

Crawley et al. [18] record that one of the largest effects of fertilization at Park Grass was the effect on soil pH and its subsequent effects on species richness (see also [9, 15]) and on the biomass of individual species [15]. We too found a significant positive relationship between species richness and pH. Like Crawley et al. [18] in the Park Grass experiment, we found that this relationship was better explained by a piecewise regression (Fig 5) than by a linear relationship. The most noteworthy effect on soil pH was due to the effect of liming. Storkey et al. [27] found that the number of species occurring on plots that stopped receiving N fertiliser in 1989 'bounced back' from the negative effects of N fertilization, which was facilitated by liming. Those Park Grass plots that stopped receiving inorganic nitrogen fertilizer in 1989 could recover much of the diversity that had been lost by 2012 once fertilization stops. These authors found no evidence that long-term nitrogen fertilization caused a reduction in species richness at Park Grass. The exception to this generalization was that, where there had been extreme acidification at Park Grass by nitrogen fertilization, such as fertilization with ammonium sulphate, species richness remained low. We found a very similar result (e.g. Fig 4). However, at Ukulinga, the loss of species was prevented (or not as many species were lost) due to lime application but did not ‘bounce back’ in the same way as at Park Grass (application of nitrogen has not been terminated at Ukulinga). Whether it would simply take more time for such replenishment of species at Ukulinga as at Park Grass to occur is unknown, although we note that Storkey et al. [27] had observed their effect over a 23-year period between the termination of inorganic nitrogen fertilization in 1989 and 2012, while our study has been running for far longer (65 years) and application of nitrogen has not been terminated.

### Responses of individual species to fertilizers, soil and light variables

In six of the seven most-abundant species, there was a significant difference in their responses to form of nitrogen fertilizer. In four of the seven species, there was a significant difference in their response to level of nitrogen fertilizer. In six of the seven species, there was a significant effect of phosphorus fertilization. These all point to the positive (additive) effect of nitrogen and phosphorus fertilization (Table 2); however, only two of these species (*E*. *curvula* and *P*. *maximum*) displayed a significant nitrogen X phosphorus interaction. However, when we tested the effect of the current level of total soil nitrogen, there was only a significant effect for *T*. *triandra* and *T*. *leucothrix*. The biomass of both of these species were negatively correlated with nitrogen, indicating that they were nitrophobic. Contrastingly, despite the fact that *P*. *maximum* is renowned for being a nitrophilic species (it often grows vigorously under nitrogen-fixing legumes such as *Acacia* tree species [83, 84]), this species did not show any significant relationship with nitrogen.

In four of the seven species (*P*. *maximum*, *T*. *triandra*, *S*. *sphacelata* and *E*. *curvula)*, there was a significant effect of lime application, which is consistent with an overall response to changes in pH (Table 4a). Fynn and O’Connor [2] also found that many individual species responded to lime application at Ukulinga, suggesting sensitivity to soil pH. We found that *S*. *sphacelata* and *T*. *triandra* responded positively to increases in pH (i.e. they responded to a more alkaline soil), while *P*. *maximum* and *E*. *curvula* responded negatively to pH, indicating that they preferred a more acidic soil. *P*. *maximum* did not respond as expected to increased total soil nitrogen (Table 4a), although it did respond to long-term nitrogen fertilization (Table 3). It appears rather that it responds to the greater acidity in the soil that is induced by nitrogen fertilization. A positive response to lime application was very similar to that found with *Arrhenatherum elatius* and *Holcus lanatus* in the Park Grass experiment [15], where liming ameliorated the effect of nitrogen fertilization on species richness due to its effects on soil pH. A similar result was reported in later studies [18, 27].

The effects of light, induced by shading among plants, appears to be important among many of the abundant species. Three of the four abundant species we studied (*E*. *curvula*, *E*. *plana*, *A*. *junciformis*) responded positively to PAR. Only one species (*Panicum maximum*) responded negatively to PAR. This means that only three species were unresponsive to PAR (*T*. *triandra*, *T*. *leucothrix*, *S*. *sphacelata*). However, there was no overall (significant) response of ANPP or species richness to PAR. Light conditions beneath the grass layer were reduced by nutrient addition to 30% of full sunlight but remained above 60% in non-fertilized plots [46]. In the Park Grass experiment, Crawley et al. [18] also found shading to be important. This may be a general pattern: Borer et al. [39] consider shading to be a common factor in many of the grasslands they have studied across the world in their grassland fertilization (Nutrient Network) experiments. Borer et al. [39] found that nutrients increase productivity, thereby reducing light availability. Herbivores reduce competition for light by removing biomass, leading to a general conclusion of light limitation at high productivities.

A very interesting result in this study was that, while there were many variables that the dominant species responded to (Tables 3 and 4a), when species richness (and ANPP in the case of *P*. *maximum*) was also introduced into the multiple regression models, these were more important than the other variables in five of the six species (Table 4b). Even for those species where there was a significant effect of pH (*T*. *leucothrix*, *S*. *sphacelata*), total soil nitrogen (*S*. *sphacelata*) and PAR (*P*. *maximum*), the standardized (β) coefficients showed that species richness was more important than the other variables. However, the directions of the effects differed among species–*T*. *triandra*, *T*. *leucothrix* and *S*. *sphacelata* responded positively to species richness while *E*. *curvula*, *E*. *plana* and *P*. *maximum* responded negatively. Some researchers have found that differences in plant height affect responses to species richness (e.g., [2, 46, 55, 72, 85]). However, we note that the tall species did not necessarily respond positively to species richness–*S*. *sphacelata* and *P*. *maximum* are tall species but one (*S*. *sphacelata*) responded positively to species richness while the other responded negatively. *T*. *triandra T*. *leucothrix*, *E*. *curvula* and *E*. *plana* are short species and they responded positively (*T*. *triandra*, *T*. *leucothrix*) and negatively (*E*. *curvula*, *E*. *plana*) to species richness. Our results show that *T*. *triandra*, *T*. *leucothrix* and *S*. *sphacelata* are facilitated by the presence of other species, while *E*. *curvula*, *E*. *plana* and *P*. *maximum* compete with other species.

An interesting species is *Aristida junciformis*, which is known to take over grasslands very quickly when they are disturbed in any way [5, 86]. From Table 4b, we see that it did not respond significantly to any of the variables, yet one might expect from the population dynamics of this species [5, 86] that it might respond to species richness. *A*. *junciformis* should be a very effective competitor. We found that *A*. *junciformis* was a very poor competitor [5]. However, if *A*. *junciformis* exploits gaps where other species are absent, a significant negative correlation with species richness and ANPP might be expected. *A*. *junciformis* is known to be very unpalatable, largely due to its high fibre content [87, 88]. Morris and Tainton [86] found that *A*. *junciformis* was more sensitive to defoliation (the grass is more palatable when it is young) than the highly palatable dominant species in many natural grasslands, *T*. *triandra*. Morris and Tainton [86] found that competition reduced yield to a greater extent in *T*. *triandra* than in *A*. *junciformis*. The two species differed in their ability to tolerate defoliation under competition. Defoliation reduced the yield of *A*. *junciformis*, at all levels of competition, to a greater extent than *T*. *triandra*. *A*. *junciformis* was particularly sensitive to a combination of severe defoliation and full competition [86]. In this study, we found that there was a significant effect of phosphorus and a significant phosphorus X lime interaction (Table 2). Furthermore, there was a significant positive effect of PAR (Table 4a). While most of the other abundant species were significantly affected by species richness and/or ANPP (Table 4b), *A*. *junciformis* did not respond to either of these parameters. Morris and Tainton (86) suggested that manipulation of grazing patterns may shift the competitive balance between palatable (e.g. *T*. *triandra*) and unpalatable (e.g. *A*. *junciformis*) components of the sward. We suggest that simultaneous manipulation of phosphorus, lime, light, competition and defoliation is needed to better understand the dynamics of *A*. *junciformis*.

## Conclusions

We believe that there clearly are generalities that can be derived from such long-term fertilization experiments. The majority of the results from this study are consistent with those of the Park Grass experiment [9, 14, 15, 18]. Specifically, we found an additive effect on ANPP of nitrogen and phosphorus fertilization, a negative effect on species richness of nitrogen fertilization, a greater negative effect on species richness of the acidic ammonium sulphate than the nitrate fertilizer, a significant positive response of species richness to pH, a positive response of species richness to lime, and a range of responses of individual species that were consistent with similar results on unrelated grass species in the Park Grass experiment. We also found a negative relationship between species richness and ANPP, as was found at Park Grass [15, 18]. In sum, the similarities between the VFE at Ukulinga and the Park Grass experiment are quite remarkable, especially in light of the differences in their climates (Table 1).
